# Care-giver identity impacts offspring development and performance in an annually social bumble bee

**DOI:** 10.1186/s12862-021-01756-2

**Published:** 2021-02-09

**Authors:** Claudinéia P. Costa, Kaleigh Fisher, Blanca M. Guillén, Naoki Yamanaka, Guy Bloch, S. Hollis Woodard

**Affiliations:** 1 Department of Entomology, University of California, Riverside, CA USA; 2 Department of Ecology, Evolution and Behavior, Alexander Silberman Institute of Life Sciences, Hebrew University of Jerusalem, Jerusalem, Israel

**Keywords:** Bumble bees, Maternal influence, Offspring development

## Abstract

**Background:**

The developmental fates of offspring have the potential to be influenced by the identity of their care-givers and by the nature of the care that they receive. In animals that exhibit both parental and alloparental care, such as the annually eusocial insects, the influence of care-giver identity can be directly assessed to yield mechanistic and evolutionary insights into the origins and elaboration of brood care. Here, we performed a comparative investigation of maternal and worker brood care in bumble bees, a pollinator group where mothers (queens) rear the first offspring in the nest, and then daughters (workers) assume this role upon their emergence. Specifically, we compared the effects of queen and worker brood care on offspring development and also offspring performance, for a set of traits related to sensory biology, learning, and stress resistance.

**Results:**

We found that queen-reared workers were smaller-bodied than worker-reared offspring, suggesting that bumble bee queens influence body size determination in their offspring. We also found that queen-reared workers were more resistant to starvation, which might be beneficial for early nesting success. These maternal influences could not be explained by feeding rate, given that we detected a similar offspring feeding frequency in both queens and workers.

**Conclusion:**

Bumble bee queens have a unique influence on the development of the first offspring in the nest, which they rear, relative to worker-reared workers. We propose that bumble bee brood care has been shaped by a suite of evolutionary and ecological factors, which might include a maternal influence on traits that promote survival of incipient colonies.

## Background

Extended parental care is widespread in the Animal Kingdom, but is relatively rare in insects, where it has evolved in some groups such as burying beetles, earwigs, and several lineages in the order Hymenoptera [1–3]. The eusocial insects express an additional, alloparental form of care, in which some daughters remain in nests as workers and care for their young, developing siblings. In these systems, workers are largely sterile caregivers [4]. Theoretical explanations have been developed for the evolution of brood care. Parental investment is predicted to evolve when it increases parental fitness, even if future reproductive costs are incurred [5–8], whereas the evolution of sibling care can be explained by the benefits it confers to individuals or social groups, which can be explained by inclusive fitness or multi-level selection theory [9-11; reviewed by 12]. However, despite these theoretical advances, improving our understanding of the origins, mechanistic basis, and consequences of brood care remains a key goal in social insect research [13].

A small subset of eusocial insect species exhibit both maternal and sibling care, albeit at different life history stages. This is seen in the eusocial species where nests are founded by a solitary queen, which includes some primitively eusocial bees, including bumble bees (genus *Bombus*, family Apidae), and others hymenopterans, such as paper wasps (genus *Polistes*, family Vespidae) and the solitary founding ants. In these systems, queens care for their brood during initial stages of colony development, then cease providing care around the time that workers eclose in the nest and begin to care for their siblings [14–17]. Solitary founding queens face unique challenges at the nest-founding stage, as they physiologically prepare for the onset of egg-laying, tend to the nest, and then care for offspring as they develop. At this stage, the success of the nest rests entirely on a single individual, the queen. In species that found nests in spring, such as bumble bees, climate change-driven mismatches with food resource availability can be more detrimental at this stage relative to later in the nesting season [18, 19]. Nests are also subject to other stressors, such as pesticides, and many solitarily-founded nests likely fail at the initiation stage, which precludes the production of new reproductives (new queens and males) [20, 21].

Many forms of brood care include progressive provisioning [22], which refers to the continuous feeding of larvae by adults, usually through close contact of the mouthparts and regurgitation of food. This dynamic social feeding interaction is a nexus of cooperation and conflict between provisioning adults and brood. Developing larvae can produce signals that elicit regurgitation by adults [23, 24]; produce priming pheromones that stimulate foraging activity [25, 26]; and communicate information to provisioning adults about nutritional status and needs [23, 27, 28]. These larval hunger signals can be honest signals that reflect nutritional needs or they can be manipulative and uncoupled from nutritional state [24]. Progressive provisioning also creates an opportunity for the manipulation of offspring by care-givers. Food amount and composition are key determiners of adult body size and direct fitness in insects [29–31]. In social insects, larval diet can also influence female caste determination [32, 33]. Thus, the transition to producing new queens in the nest is partially under the control of those who feed brood, and its timing subject to evolutionary pressures related to queen-worker conflict [34–36]. However, in the young nests of solitary-founding queens, where workers have not yet emerged, larval feeding might be more directly limited by the amount of feeding that individual queens can provide.

Here, we report results of an experimental study on the development of the first brood in the bumble bee *Bombus impatiens*. We performed an experiment to determine how queen and worker brood care uniquely impact the developmental fates of offspring, by manipulating young nests such that the first cohort of female brood was either reared solely by a queen or by a small cohort of workers. We then examined how care-giver identity impacted offspring developmental duration and body size. We predicted that queen-reared workers would be fed less frequently and be smaller-bodied, based on the hypothesis that bumble bee queens are limited in the amount of brood care that they can provide, relative to a group of workers. This prediction was also based on a previous study in *Bombus terrestris* that found that female brood reared by the queen develop for a shorter duration and may be smaller than worker-reared offspring [14]. We also explored differences in the quality or performance of adult offspring reared by either a single queen or a set of workers, for a set of traits related to sensory biology, learning, and stress resistance. The three traits that we examined are each intricately linked to colony development and survival. Sucrose sensitivity and learning can be associated with foraging specialization [37, 38] and efficiency [39], and thus have implications for colony success, whereas resistance to starvation might allow colonies to survive during periods of nutritional stress [40]. Here, our predications were based on the hypothesis that rearing by the queen may have been influenced by selective pressures related to the challenges of solitary nest founding. Specifically, we predicted that we would observe characteristics that are beneficial for early nest establishment in queen-reared workers, such as improved learning abilities and greater resistance to starvation. With respect to sucrose responsiveness, we predicted that queen-reared workers would possess lower response thresholds. This prediction was based on a previous study that found that in honey bees, workers with lower sucrose response thresholds prioritize pollen collection over nectar [41]. If this is also true in bumble bees, such a bias might be advantageous for increasing early colony growth rates [42] and improving the likelihood of nesting success.

## Results

### Influence of care-giver identity on offspring developmental duration and body size

**Fig. 1.**
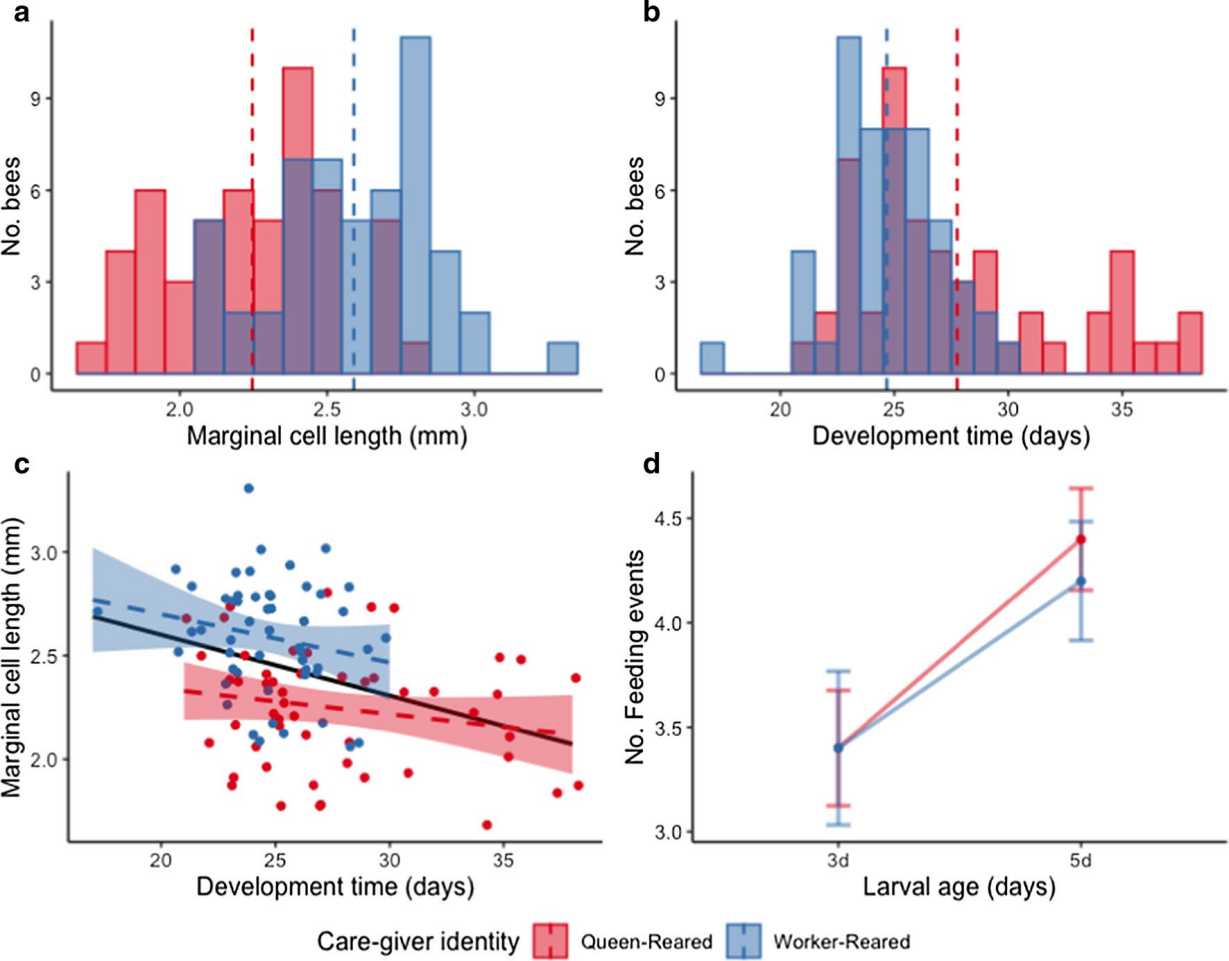
Effects of care-giver identity on body size, developmental duration, and feeding events. Young nests were manipulated such that the first cohort of female brood was either reared solely by a queen (QR nests) or by a small cohort of five workers (WR nests). **a** Body size as a function of care-giver identity; x-axis, length of the second marginal cell of the wing as a proxy for body size; y-axis, total number of bees; dashed lines represent the mean body sizes for each group (QR nests: mean ± s.e.m. 2.25 ± 0.03 mm; WR nests: 2.59 ± 0.02 mm). Care-giver identity impacted body size (Tukey’s post doc: QR versus WR: *p* < 0.001). **b** Developmental duration as a function of care-giver identity; x-axis, egg to adult development duration; y-axis, total number of bees; dashed lines represent the mean development durations for each group (QR nests: mean 27.75 ± 0.12 s.e.m. days; WR nests: mean 24.67 ± 0.06 s.e.m. days). Care-giver identity did not impact developmental durations. **c** Relationship between body size and developmental duration; dashed lines represent regression lines for each group and the solid line represents the regression line for all samples combined. There was a significant negative correlation between bee body size and developmental duration (rho = − 0.328, *p* < 0.001). **d** The total number of brood-feeding events exhibited, as a function of nest type; x-axis, day after larvae were first present in the nest (e.g., larval ages of 3 and 5 days); y-axis, number of brood-feeding events observed per day (mean ± s.e.m), for a total of 60 minutes of observation per day. Values are not corrected by the number of larvae in the nest, but these are assumed to be ~5 in all nests (based on Ref. 81 and S. H. W., unpublished data). Comparison performed by one-way analysis of variance (ANOVA) groups, care-giver, (N.S.).

We generated sets of nests that contained brood reared either solely by the queen (hereafter, queen-reared or “QR” nests; *n* = 13) or by workers (worker-reared or “WR” nests;* n* = 9). Next, we compared offspring developmental durations (time from egg laying until adult eclosion) and body sizes (estimated from the lengths of the second marginal cells of the wings) for these nests (*n* = 104 bees total from 22 nests). The best-fit model explaining body size included care-giver identity as a fixed effect [GLMM: significant group term (QR and WR), comparison of the model and null model: LRT: χ^2^ = 8.629, d.f. = 1, *p* = 0.003]; Table 1. Offspring reared solely by a queen were significantly smaller than those reared by a group of five workers (mean ± s.e.m. marginal cell length of 2.25 ± 0.03 mm versus 2.59 ± 0.02 mm, respectively; Tukey’s post doc: QR versus WR treatment groups: *p* < 0.001); Table 1; Fig. 1a. Care-giver identity did not impact developmental durations (Table 1; Fig. 1b). Body sizes and developmental durations were negatively correlated (Spearman: rho = -0.328, *p* < 0.001; Fig. 1c). One gyne pupal cell (determined based on its size) was observed in a worker-reared nest. This gyne was not included in any statistical analyses because the developmental patterns of queen and worker larvae are not comparable with respect to developmental duration or adult body size [43, 44].

**Table 1.** Predictors of offspring body size and developmental duration

**Trait**	**Analysis**	**Model**	**Estimate**	**Standard error**	**z-value**	***p-value***	**Direction**
Body size	Gamma GLM	Body size ~ Group (care-giver) + random factor “source colony” + random factor “nest ID”	0.444	0.134	z = 3.312	< 0.001 ***	QR < WR
Developmental duration	Gamma GLM	Development time ~ + random factor “source colony” + random factor “nest ID”	na	na	na	na	na

Results are from best-fitting models. The Generalized Linear Model (GLM) for Gamma distribution was used in modeling continuous data. Asterisks indicate statistical significance; significance code: ‘***’ 0.001

### Differences in queen and worker brood-feeding behavior

We compared the number of brood-feeding events exhibited in a subset of queen-reared versus worker-reared nests (*n* = 5 nests per group). There was no difference in the number of feeding events performed in QR nests (mean ± 1 s.e.m. events = 3.4 ± 0.28 and 4.4 ± 0.24 for days 3 and 5, respectively) and WR nests (3.4 ± 0.37 and 4.2 ± 0.28 for days 3 and 5, respectively) during the 120 min of observation per nest (ANOVA: F = 0, *p* = 1 and F = 0.067, *p* = 0.803 for days 3 and 5, respectively; Fig. 1d).

### Influence of care-giver identity on offspring sucrose responsiveness

We then subjected a randomly selected subset of bees (adult age three days) from the QR (*n* = 25) and WR (*n* = 26) nests (from 11 and 8 nests, respectively) to a sucrose responsiveness assay. Care-giver identity did not impact whether or not bees responded to sucrose, at any concentration, or sucrose response threshold (Table 2; see Additional file 1: Fig. S3). The best-fit model for sucrose responsiveness included developmental duration as a predictor variable, but this predictor did not significantly impact sucrose responsiveness when the model was employed (GLMM: no significant development duration term; comparison of the model with factor versus null model: LRT: χ^2^ = 3.831, d.f. = 1, *p* = 0.051); Table 2.

**Table 2.** Predictors of offspring performance, including sucrose responsiveness, sucrose concentration, color training, and starvation resistance

**Assay**	**Analysis**	**Model**	**Estimate**	**Standard error**		**Test statistic**	***p-value***	**Direction**
Sucrose responsiveness	Binomial GLM	Yes/No (1/0) ~ Development time + random factor “source colony” + random factor “nest ID”	0.198	0.105		z = 1.875	0.061	Longer > Shorter durations
Sucrose concentration	Gamma GLM	Concentration response ~ + random factor “source colony” + random factor “nest ID”	na	na		na	na	na
Color training	Binomial GLM	Yes/No (1/0) ~ Body size + random factor “source colony” + random factor “nest ID	2.200	1.289		z = 1.706	0.088	Large > Small
Color learning	Binomial GLM	Yes/No (1/0) ~ + random factor “source colony” + random factor “nest ID”	na	na		na	na	na
Starvation resistance	Gamma GLM	Response (hours) ~ Group (care-giver) + Body size + random factor “source colony” + random factor “nest ID” + assays	− 0.185 0.149	0.071 0.082		z = − 2.602 z = 1.815	0.009 ** 0.069	QR > WR Large > Small

The Generalized Linear Model (GLM) for Binominal distribution was used in modeling data that had two possible outcomes (success or failure); and GLM for Gamma distribution was used in modeling non-integer data. Asterisks indicate statistical significance; significance code: ‘**’ 0.01

### Influence of care-giver identity during development on adult color training and associative learning

We also subjected a subset of offspring from QR (*n* = 27) and WR (*n* = 26) nests (from 11 and 8 nests, respectively) to an assessment of color learning. This set of offspring was different from the set of offspring used for the sucrose responsiveness assays. Care-giver identity did not affect either color training or learning (Table 2; see Additional file 1: Fig. S4). The best-fit model for the response of successful color training included body size as a predictor variable, but body size did not significantly impact color training when the model was employed (GLMM: no significant body size term; comparison of the model with a null model: LRT: χ^2^ = 3.261, d.f. = 1, *p* = 0.071); Table 2.

### Influence of care-giver identity on offspring starvation resistance

**Fig. 2.**
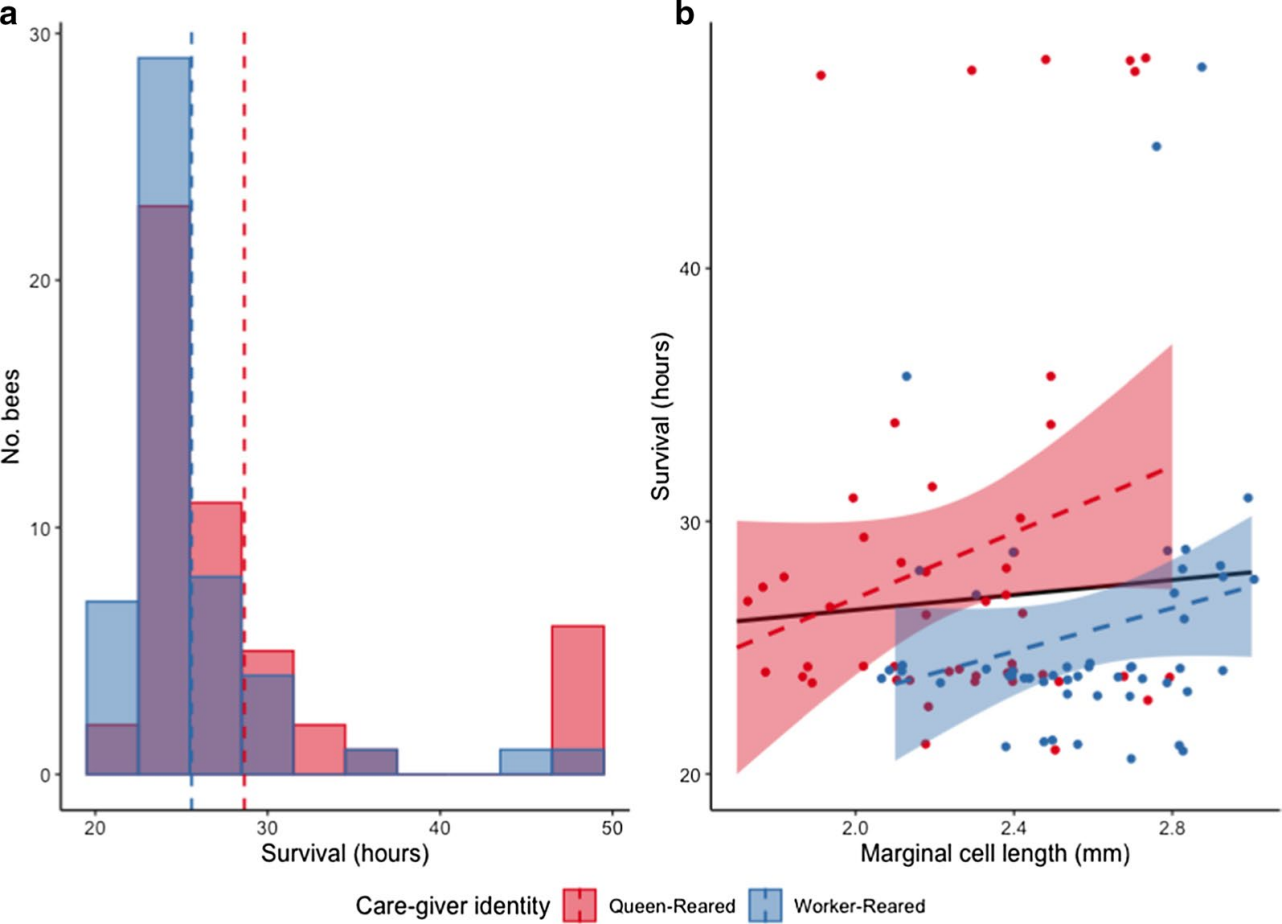
Effects of care-giver identity and body size on starvation resistance. Offspring reared by a queen (QR nests) or a small group of workers (WR nests) were subjected to assay where food was removed and time until death (starvation) was recorded. **a** Starvation resistance as a function of care-giver identity; x-axis, time until starvation; y-axis, total number of bees. Dashed lines represent the mean survival hours for each group, care-giver (QR nests: mean ± s.e.m. 28.66 ± 0.21 h; WR nests: 25.60 ± 0.14 h). Care-giver identity impacted starvation resistance (Tukey’s post doc: QR versus WR: *p* = 0.009). **b** Relationship between starvation resistance and body size (rho = − 0.056, *p* = 0.580). Dashed lines represent regression lines for each group and the solid line represents the regression line for all samples combined.

Following the sucrose responsiveness and color learning assays, workers subjected to either of these tests (*n* = 104) were examined for their ability to withstand starvation. Here, in our GLMM, body size and care-giver identity were both predictors of starvation resistance in the best-fit model, but only care-giver identity significantly impacted this variable [GLMM: no significant body size and significant group (QR and WR) terms; comparison of the model with and null model: LRT: χ^2^ = 6.179, d.f. = 2, *p* = 0.045]; Table 2. Specifically, offspring reared by queens survived the longest durations under starvation conditions (mean hours ± s.e.m. of 28.66 ± 0.21 for QR and 25.61 ± 0.14 hours for WR; Tukey’s post doc: QR versus WR: *p* = 0.009; Fig. 2a). The maximum amount of time a bee survived during the assay was 72 hours (*n* = 2); these two outliers (from one QR and one WR nest) and another one outlier based on interquartile range (from a WR nest) were removed from the analyses. Time until death and body size were not correlated (Spearman: rho = -0.056, *p* = 0.580; Fig. 2b).

## Discussion

The evolution of extended parental care and cooperative alloparental brood care are foundational for understanding the evolution of sociality [1–13]. In the subset of eusocial insect lineages that express both of these forms of care, they can be directly compared to explore their unique mechanistic bases [15–17]. Here, we show that in the bumble bee *B. impatiens*, queen and worker brood care uniquely impact the developmental trajectory and traits of offspring. Queen-reared workers in our study were smaller-bodied and also able to survive for a longer period under starvation conditions. These findings could not be explained by limitation in the frequency of brood feeding by queens, given that we detected no differences in the feeding rates of queen and worker care-givers.

We propose that our finding that queen-reared workers are more resistant to starvation might be related to the action of selection favoring worker traits that are beneficial during the early nesting period, a time when nests might be sensitive to food shortages [42] and when many wild bumble bee nests likely fail [20, 21, 45]. Annually social (or recently-formed) societies might experience intense selection for the first brood to be particularly capable of carrying out tasks related to nest development and survival. This is because the success of nests rests more heavily on these early emerging individuals, versus later in colony development when the social group is larger and there are more individuals present to carry out tasks [46]. For social species that initiate nests in early spring (including bumble bees), the first offspring may also need to withstand periods of nutritional dearth that are longer or more frequent. Floral food resources can be less consistent early in the season in some environments [18, 19], and fluctuating weather regimes may make it impossible to forage at times [47]. Further, food stores are less abundant at this stage in bumble bee colony development [47], and even mature colonies typically contain only enough stores to survive up to a few days without replenishment [47, 48]. In previous studies of *B. impatiens*, Couvillon and Dornhaus [40] found that smaller-bodied workers are also hardier against starvation in mature colonies, in part because they have higher lipid levels than larger-bodied workers [49; but see 50]. Our finding that queen-reared workers are more resistant to starvation, irrespective of body size, suggests that a complex suite of factors control this trait in workers, and that these factors potentially change across the course of bumble bee colony development [40, 49, 50].

Our finding that queens rear smaller-bodied offspring than workers is consistent with a previous study in the bumble bee *B. terrestris*, which found that rearing by the queen results in a shorter developmental duration in workers, and can cause a decrease in body size, during the establishment period of newly-formed nests [14]. The maternal manipulation hypothesis for the evolution of sociality posits that maternal control of direct fitness outcomes of offspring (in part through feeding and diet) played a role in the origins of social life, such that offspring were manipulated to remain within their natal nests as helpers, rather than leave and initiate their own nests [51, 52]. One of the key predictions of this hypothesis is that mothers should rear female daughters that are smaller or otherwise less able to successfully carry out nesting on their own [51]. Evidence for maternal manipulation has been found in a number of insect lineages, including weakly or flexibly social bees [53–56], and in many vertebrate species [e.g., 57,58]. In eusocial insects, maternal manipulation theory has been extended to include caste bias, such that in the systems with both maternal and alloparental care, queens are predicted to rear offspring that are more likely to develop into sterile workers versus reproductive queens [59]. This extension of maternal manipulation theory was developed because eusocial insect workers do not require manipulation to remain as helpers in the nest, but development into a queen might allow an individual to leave and initiate a new nest. This theoretical prediction has been met in paper wasps [59], and was not explicitly met in our study, because we saw smaller-sized workers in our queen-reared, but did not see more gynes.

As an alternative to the maternal manipulation hypothesis, the smaller body sizes we observed in queen-reared workers might be more related to colony efficiency, or some other beneficial outcome of having smaller-sized workers in young nests. In bumble bees, there is considerable variation in worker body sizes within mature nests (reviewed in ref. 33), which is considered adaptive in that it contributes to social organization and overall colony success [60]. Larger workers forage at an earlier age and are more frequent [61, 62] and efficient [63–65] foragers. They have a greater density of olfactory sensilla and sensitivity to odorants [66], which may impact learning, and are more positively phototactic [67], which is consistent with their increased propensity to forage. However, smaller workers are more likely to be found in the nest, are more resistant to starvation in previous studies [40], and are more likely to incubate brood [68]. Collectively, the maintenance of this worker variation in colonies is thought to improve colony efficiency [69]. However, empirical studies have shown that colonies can perform equally successfully when they are comprised of skewed or more limited worker body size distributions [69] and may produce skewed size distributions under some conditions, such as when resources are plentiful [42]. Based on our current understanding of bumble bee biology, the significance of having smaller-bodied workers in young, recently-founded nests is unclear. Our finding that variation in associative learning and sucrose sensitivity could not be attributed to body size (or care-giver identity) suggests that any benefit of smaller-sized workers is not related to these specific cognitive and sensory traits in *B. impatiens.* However, given that size-related differences in learning abilities have been detected in other studies [70–72], we suggest that further studies are required in this avenue of research. Additionally, further explorations of how maternal and sibling care shape worker characteristics, and the adaptive benefits of reduced body sizes in young nests, are important for fully understanding the functional and evolutionary significance of the patterns that we have detected.

## Conclusions

Here, we examined how brood care by a queen or workers impact offspring development in a bumble bee species, and found distinct differences in how these two forms of care influence offspring size and resistance to starvation. We observed that queen-reared larvae were fed no less frequently, as has also been reported for *B. terrestris* [14]. Given this, there may be other differences in the quality or nature of maternal and sibling care in bumble bees, such as the quantity of food delivered per feeding event or the composition of larval food [73], or in non-nutritive signals such as contact or volatile pheromones [14] or the frequency or quality of brood incubation [74]. Thus, a critical remaining question is how queens influence offspring development. Broadly, our study provides additional evidence that maternal interactions can influence early development [75, 76], including in eusocial lineages [59]. Further, despite their phenotypic similarity, maternal and sibling care appear to have been subject to unique selective pressures during the evolution of the bumble bee lineage.

## Methods

### Bee rearing and behavioral observations

All bees originated from mature colonies (queenright with > 50 workers) supplied by Koppert Biological Systems (Howell, MI, USA). We housed these source colonies in the Entomology Building at the University of California, Riverside, maintained at room temperature (~23°C) and uncontrolled (but > 40%) RH. Colonies were fed a syrup solution (provided with colonies by Koppert Biological Systems) and mixed-source, honey bee-collected pollen (Biobest USA, Inc.) provided *ad libitum*. All individual queens and nest cages were kept at the University of California, Riverside’s Insectary and Quarantine Facility in a room maintained at 27-30°C and 72-80% RH. Individual bees were fed the same pollen diet described above and an artificial nectar solution based on [77].

Nests were created to obtain brood reared solely by a queen (hereafter, queen-reared or “QR” nests; *n* = 13) or by a set of five workers (worker-reared or “WR” nests;* n* = 9). To create these two nests types, first, newly eclosed (callow) queens were removed from their natal colonies and mated with males from different source colonies, based on methods in Röseler [78], with the following differences. Callow queens (< 24 hours old; identified by their silvery appearance and inability to fly) were placed in individual plastic queen rearing cages obtained from Biobest USA, Inc. (approximately W15 x D15 x H10 cm). At ages 3-8 days, the queens were placed in mating cages (W60 x D60 x H120 cm; BugDorm-6S620) from approximately 900-1700 hours with males (2-3 per queen). After mating once [79, 80], queens were placed in new plastic rearing cages (as described above). At ages 12 and 13 days, queens received a CO_2_ treatment (30 minutes per day) to stimulate nest initiation [78]. All queens used in the experiment initiated nests within a mean (± 1 s.e.m.) of 16 ± 1.59 days after the CO_2_ treatment.

Five days after eggs were first observed in a nest (the time until eggs become larvae; ref. 44), queens were either left in their cage (for QR nests) or were removed and five callow workers were added (WR nests). This is the average number of eggs that a queen lays in her first egg cell, and thus the average number of individuals in the first brood in *B. terrestris* [81] and *B. impatiens* (unpublished data, S.H.W.). All workers introduced to a nest came from the same source colony, which differed from the queen’s natal colony. To ensure that the correct ages of eclosed adults were determined, we destroyed any additional eggs laid on the following five days after the first set of eggs were detected. This is conservative in that if it was unclear whether a larva originated from the first brood cohort or not, it was destroyed. This allowed us to have high confidence in brood ages, at the cost of likely reducing the number of brood obtained from some nests. All data reported hereafter are related only to the first brood cohort, which consisted solely of female brood; no male offspring were observed in these nests or included in our analyses. We placed infrared security cameras (VIGICA Peashooter QD520) above both the QR and WR nests for the duration of the experiment, which was done to generate video data for estimating feeding frequency and the time of eclosion for all offspring (see below). Nests were inspected daily and the dates of adult emergence were recorded. No queens died during the experiment. Any adult workers (i.e., care-givers) that died (*n* = 4) were replaced with callow workers from the same source colony.

### Developmental duration and body size

To quantify the adult (final) body size of offspring, we measured the length of the second marginal cell of the wing for all offspring from the first brood cohorts in our QR and WR nests (*n* = 104 bees). This metric is highly correlated with body size in bumble bees [14, 61, 82, 83]. To quantify developmental duration for all offspring (*n* = 104 bees), we calculated the number of days between when eggs were laid until adult eclosion. This duration was determined based on video data obtained from infrared security cameras.

### Estimation of feeding frequency

To estimate larval feeding frequency in our QR and WR nests, we selected a subset of five nests from each of the two treatment groups for observational analysis. Nests were selected for this analysis if they contained brood with the shortest or longest developmental durations, or they were randomly selected from the middle of this distribution. This was done to maximize our ability to detect differences in feeding rates between the two nest types. Brood-feeding is a discrete behavior that involves piercing the larval wax envelope, placing mouthparts through the opening, and regurgitating with an abdominal contraction [81] (see video in Dryad). For each nest, we observed five minutes per hour within a 12-h period on day 3 and again on day 5 after the first larvae were present in the nest, for a total of 120 min of observation per nest. This amount of observation was based on previous studies that examined brood-feeding behavior in bumble bees [14, 81]. A single observer viewed videos to avoid introducing observer bias. In our results, we show the rate of feeding events performed by a single queen or by a group of workers, not corrected by the number of larvae in the nest. Here we assume that there were equal numbers of larvae (~5; ref. 44) in all nests irrespective of nest type, given that the number of eggs in the first cohort was fixed before nests were assigned to a specific treatment group.

### Sucrose responsiveness assay

On the third day after adult emergence, bees were removed from the nest and placed individually in a 14 ml centrifuge tube, modified to allow the worker to receive taste stimulation (modeled after [84]). Bees were starved of pollen and artificial nectar in these tubes for 3-5 hours prior to the assays. Then, we dipped a wooden toothpick into sucrose solutions at one of the following concentrations (w/v) and touched the antennae to attempt to elicit a proboscis extension reflex (PER): 20%, 25%, 30%, 35%, 40%, 45%, 50%. We administered concentrations in the order listed, with deionized water used as a negative control in between each concentration. No workers responded to this negative control. We used higher sucrose concentrations than are used for honey bee PER [85] (as have been used before in bumble bees, refs. [86, 87]) because in preliminary tests, we found that 20% sucrose was the lowest concentration that elicited a response. As a response variable (sucrose responsiveness), we recorded the minimum concentration at which a worker extended her proboscis for at least three seconds. Bees that respond to lower sucrose concentrations have a lower sucrose response threshold and are more sensitive to sucrose. We also considered whether or not a bee responded at all to sucrose (at any concentration) during this assay, as some bees (*n* = 22) did not. During the assay, bees were kept under controlled conditions in the insectary room described above. No bees died during this assay.

### Color learning assay

To assess learning ability, we removed individual bees from their nests on the third day after adult emergence and subjected them to an assay based on [88]. During this period, individual bees were maintained in the insectary room described above. Workers were starved of pollen and artificial nectar for 3-5 hours prior to the assay in a 5 ml tube with holes for ventilation and two holes at the end to insert test strips. Our learning assay consisted of two phases: (1) color training and (2) color learning. During the training phase, bees were either given a yellow or blue strip of paper coated with 50% (w/v) sucrose solution to train them to associate the color with sucrose. Workers were then given a maximum of five minutes to respond to the stimulation and a response was recorded when a worker extended her proboscis and made contact with the paper for at least three seconds. After a successful response, the paper strip was removed from the tube and the bee was undisturbed for five minutes. This was repeated for a total of five times, alternating the hole that the paper strip was presented in to avoid spatial learning, and with a five-minute rest period between each stimulation. Here, our response variable was whether the bee successfully extended her proboscis to the sucrose coated strip each time (positive) or not (negative). Bees that did not respond successfully five times were marked as not being trained. We consider this training component of the color learning assay to be related to the propensity to respond repeatedly to sucrose as a stimulus, and not directly related to learning ability; although, previous studies have shown an association between sucrose sensitivity and learning (reviewed in ref. 41). During the subsequent learning phase, we introduced both blue and yellow strips into the tube simultaneously (both coated in distilled water) and bees were given one minute to extend their proboscis to a test strip. We then recorded whether the bee extended her proboscis for the color paper strip that she had been trained to during the learning training phase. This second component of the learning assay is directly related to learning ability, unlike the training component. No bees died during this assay. Unique subsets off bees were used in the sucrose responsiveness and color learning assays.

### Starvation resistance assay

Following the sucrose responsiveness and color learning assays (no more than 4-5 hours after bees were removed from nests), we placed workers subjected to either of these tests (*n* = 104) in individual cages without pollen diet or artificial nectar solution in the insectary rearing room (described above). Cages were inspected at 3-h intervals from 900 to 1800 each day and the date and time that any worker was observed dead in the cage was recorded.

### Statistical analyses

All statistical analyses were performed in R version 3.6.1 [89] and only *p*-values < 0.05 were considered significant. All results were visualized with the ‘ggplot2’ package [90]. We employed the interquartile range technique and a 95% confidence interval to remove outliers, which resulted in the removal of three data points in the starvation data set (see results). After identifying non-normality of the data using the Shapiro-Wilk test, we used generalized linear mixed models (GLMMs) to explore how factors such as care-giver identity, body size, and developmental duration contributed to responses. GLMMs were performed with the glmer function in the R package lme4 version 1.1-10 [91]. The best-fit model for our data was selected based on the Akaike’s Information Criterion (AIC) using the “dredge” command within the MUMIn package [92]. Following model selection, factors of interest were analyzed by performing Likelihood Ratio Tests (LRT) comparing the models with factors to a null model without these factors. Post-hoc t-tests were conducted using Tukey’s multiple comparison of means. For responses, we considered body size, developmental duration, whether a bee responded to sucrose at all in the sucrose responsiveness assay (yes or no), sucrose responsiveness (lowest concentration a bee responded positively to), color training (whether the bee successfully extended her proboscis during training positively), color learning (bee extended her proboscis for the color paper strip that she had been trained to during the learning training phase), and survival time under starvation. Models could include as fixed effects care-giver identity, developmental duration, and/or body size, and their interactions. Queen source colony and individual nest were included as random factors in all models [92]. For starvation resistance, we included whether a bee was subjected to behavioral assays (sucrose or learning assays) as a random factor in GLMM. We used Spearman's correlation analyses (for non-parametric data) to examine correlations between body size and developmental duration, and between body size and time until starvation. A one-way analysis of variance (ANOVA) was employed to compare data on brood-feeding behavior in the nest, according to previous studies in bumble bees [14, 81].

## Supplementary Information

**Additional file 1.** Additional materials and methods.

## Data Availability

All analyses and pipelines can be found in the primary author’s GitHub (https://github.com/claudinpcosta/2021-BS.experiment-MaternalSiblingCare). Data are also available on Dryad at https://doi.org/10.6086/D1B37V [93].

## Abbreviations

QR: Queen-reared
WR: Worker-reared
