# Supplementary material for: Care-giver identity impacts offspring development and performance in an annually social bumble bee
Source: BMC Ecol Evol. 2021 Feb 9;21:20. doi: 10.1186/s12862-021-01756-2 (PMC7871553; doi:10.1186/s12862-021-01756-2)
Supplement: Supplementary file 1 — Additional file 1. Additional materials and methods. [file 12862_2021_1756_MOESM1_ESM.docx]

Supplementary Information for:

Costa CP, Fisher K, Guillén BM, Bloch G, Yamanaka N, Woodard SH. Care-giver identity impacts offspring development and performance in an annually social bumble bee

**ADDITIONAL FILES:**

All data files and a video file of feeding behaviour is provided on Dryad (<https://doi.org/10.6086/D1B37V>) and GitHub (<https://github.com/claudinpcosta/2021-BS.experiment-MaternalSiblingCare>).

**MATERIALS AND METHODS**

**(a) Additional methodological details for color learning assay**

Following [1], first, we placed bees in tubes to allow them to become sufficiently motivated to participate in the experiment; because some bees can present an aggressive behavior and affect their response on assays. For sucrose responsiveness, a small, rectangular piece of mesh was introduced and fastened to the interior of the tube using orthodontic wax in order to give support for the worker to grab on to, and be calm during the assay. The tube was then positioned in a styrofoam plate workers were encouraged to stay in place near the tip opening.

**(b) Among-nest variation in developmental duration and body size**

**
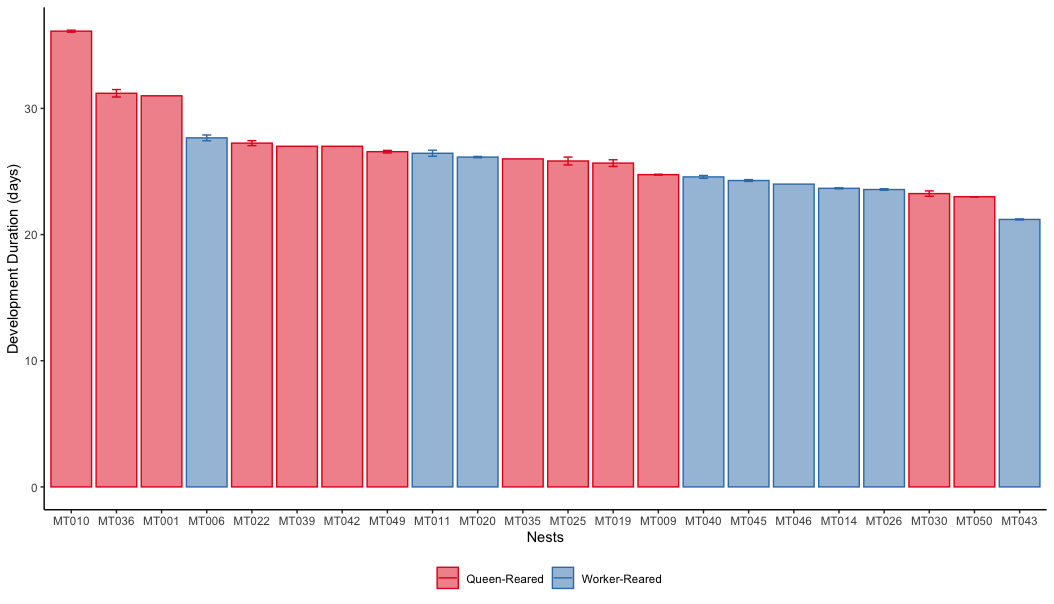
**

**Figure S1 | Development durations.** x-axis, nests; y-axis, mean (+/- s.e.m.) days from egg to adult. Young nests were manipulated such that the first cohort of female brood was either reared solely by a queen (hereafter, Queen-Reared or “QR” nests) or by a small cohort of workers (a set of five workers; Worker-Reared or “WR” nests).

**
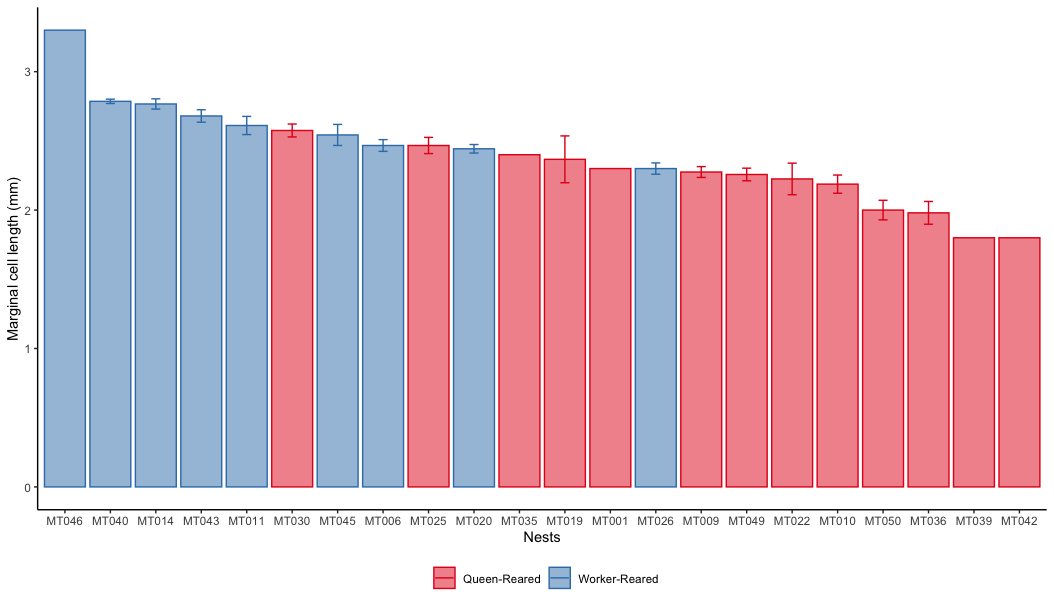
**

**Figure S2 | Body size.** x-axis, nests; y-axis, mean (+/- s.e.m.) length of the second marginal cell of the wing as a proxy for body size. Young nests were manipulated such that the first cohort of female brood was either reared solely by a queen (hereafter, Queen-Reared or “QR” nests) or by a small cohort of workers (a set of five workers; Worker-Reared or “WR” nests).

**(c) Data Analyses**

***1. Body size:*** All statistics related to model selection are summarized in Table S1.

**Table S1.** Selection of the best-fitting model explaining body size patterns. The models included rearing history (R; *i.e.,* rearing by queen or workers), developmental duration (D) as fixed factors, and source colony for queens and individual nest as the random factors. The best model fitting our data was selected based on the Akaike’s Information Criterion (AIC). (+) parameters included in the model, and NA, not included parameters. The selected best model is given in bold.

| **Body Size** | | | | | | | | |  |
| --- | --- | --- | --- | --- | --- | --- | --- | --- | --- |
| **Model** | **R** | **D** | **T:D** | **family** | **df** | **AICc** | **delta** | **weight** | |
| Null | NA | NA | NA | Gamma  (identity) | 4 | 24.2 | 6.42 | 0.022 | |
| **BS1** | **+** | **NA** | **NA** | **Gamma**  **(identity)** | **5** | **17.7** | **0** | **0.547** | |
| BS2 | + | + | NA | Gamma  (identity) | 6 | 19.3 | 1.59 | 0.247 | |
| BS3 | NA | NA | + | Gamma  (identity) | 7 | 20 | 2.31 | 0.172 | |
| BS4 | NA | + | NA | Gamma  (identity) | 5 | 25.3 | 7.54 | 0.013 | |

***2. Developmental duration:*** To determine whether there was a difference between the developmental duration as a function to rearing history (QR and WR), we carried out Gamma GLMMs (in the lme4 package; [2]) to determine whether developmental duration was determined by the rearing history (QR and WR nests). We initially ran a null model without these factors and another model including our fixed factors (the AIC of the null model was smaller than the AIC with the interest fixed factor; 476.7 compared to 477.1). For all analyses, we included colony source for queens and individual nest as the random factors.

***3. Sucrose responsiveness assay*:** To determine whether there was a difference between the sucrose response (*i.e.*, positive or negative response) as a function group (QR and WR), we carried out Binomial GLMMs with a binomial response ("positive" (1) or "negative" (0)) and the predictor factors as rearing history, body size, and developmental duration. We initially ran a null model without these factors and other models including these factors and interaction terms. Finally, we carried out Gamma GLMMs with sucrose concentration-response and possible explanatory factors (rearing history, body size, and developmental duration). For all analyses, we included colony source for queens and individual nests as random factors. All statistics for the model selection are summarized in Table S2.

**Table S2.** Selection of the best-fitting model explaining the sucrose assays. The models included rearing history (R; *i.e.,* rearing by queen or workers), body size (B), and developmental duration (D) as the fixed factors, and source colony for queens and individual nest as the random factors. The best model fitting our data was selected based on the Akaike’s Information Criterion (AIC). (+) parameters included in the model, and NA, not included parameters. The selected best model is given in bold.

| **Sucrose Response** | | | | | | | | | | |
| --- | --- | --- | --- | --- | --- | --- | --- | --- | --- | --- |
| **Model** | **R** | **B** | **D** | **S:D** | **T:S:D** | **family** | **df** | **AICc** | **delta** | **weight** |
| Null | NA | NA | NA | NA | NA | Binomial  (logit) | 3 | 75.3 | 1.47 | 0.174 |
| SU1 | + | NA | NA | NA | NA | Binomial  (logit) | 4 | 77.5 | 3.67 | 0.058 |
| SU2 | NA | + | NA | NA | NA | Binomial  (logit) | 4 | 77.7 | 3.81 | 0.054 |
| **SU3** | **NA** | **NA** | **+** | **NA** | **NA** | **Binomial**  **(logit)** | **4** | **73.9** | **0** | **0.363** |
| SU4 | + | + | + | NA | NA | Binomial  (logit) | 6 | 78.8 | 4.94 | 0.031 |
| SU5 | + | NA | NA | + | NA | Binomial  (logit) | 7 | 80.4 | 6.51 | 0.014 |
| SU6 | + | + | NA | NA | NA | Binomial  (logit) | 5 | 80 | 6.13 | 0.017 |
| SU7 | + | NA | + | NA | NA | Binomial  (logit) | 5 | 76.3 | 2.42 | 0.108 |
| SU8 | NA | + | + | NA | NA | Binomial  (logit) | 5 | 76.2 | 2.36 | 0.111 |
| SU9 | NA | NA | NA | + | NA | Binomial  (logit) | 6 | 77.7 | 3.82 | 0.054 |
| SU10 | + | + | + | + | NA | Binomial  (logit) | 7 | 80.4 | 6.51 | 0.014 |
| SU11 | NA | NA | NA | + | NA | Binomial  (logit) | 10 | 88 | 14.14 | 0 |
| SU12 | + | + | + | + | NA | Binomial  (logit) | 10 | 88 | 14.14 | 0 |
| SU13 | NA | NA | NA | + | NA | Binomial  (logit) | 10 | 88 | 14.14 | 0 |
| SU14 | + | + | + | + | NA | Binomial  (logit) | 10 | 88 | 14.14 | 0 |
| SU15 | NA | NA | NA | + | NA | Binomial  (logit) | 10 | 88 | 14.14 | 0 |
| SU16 | + | + | + | + | NA | Binomial  (logit) | 10 | 88 | 14.14 | 0 |
| SU17 | NA | NA | NA | + | NA | Binomial  (logit) | 10 | 88 | 14.14 | 0 |
| SU18 | + | + | + | + | NA | Binomial  (logit) | 10 | 88 | 14.14 | 0 |
| SU19 | NA | NA | NA | + | NA | Binomial  (logit) | 10 | 88 | 14.14 | 0 |
| **Sucrose Concentration** | | | | | | | | | | |
| **Model** | **R** | **B** | **D** | **S:D** | **T:S:D** | **family** | **df** | **AICc** | **delta** | **weight** |
| **Null** | **NA** | **NA** | **NA** | **NA** | **NA** | **Gamma**  **(log)** | **4** | **-47.2** | **0** | **0.433** |
| SC1 | + | NA | NA | NA | NA | Gamma  (log) | 5 | -45.3 | 1.9 | 0.168 |
| SC2 | NA | + | NA | NA | NA | Gamma  (log) | 5 | -44.4 | 2.83 | 0.105 |
| SC3 | NA | NA | + | NA | NA | Gamma  (log) | 5 | -44.8 | 2.46 | 0.127 |
| SC4 | + | + | + | NA | NA | Gamma  (log) | 7 | -40.6 | 6.63 | 0.016 |
| SC5 | + | NA | NA | + | NA | Gamma  (log) | 8 | -36.8 | 10.37 | 0.002 |
| SC6 | + | + | NA | NA | NA | Gamma  (log) | 6 | -43.3 | 3.95 | 0.06 |
| SC7 | + | NA | + | NA | NA | Gamma  (log) | 6 | -43 | 4.2 | 0.053 |
| SC8 | NA | + | + | NA | NA | Gamma  (log) | 6 | -41.6 | 5.56 | 0.027 |
| SC9 | NA | NA | NA | + | NA | Gamma  (log) | 7 | -38.4 | 8.85 | 0.005 |
| SC10 | + | + | + | + | NA | Gamma  (log) | 8 | -36.8 | 10.37 | 0.002 |
| SC11 | NA | NA | NA | + | NA | Gamma  (log) | 11 | -23.5 | 23.68 | 0 |
| SC12 | + | + | + | + | NA | Gamma  (log) | 11 | -23.6 | 23.62 | 0 |
| SC13 | NA | NA | NA | + | NA | Gamma  (log) | 11 | -23.6 | 23.62 | 0 |
| SC14 | + | + | + | + | NA | Gamma  (log) | 11 | -23.5 | 23.68 | 0 |
| SC15 | NA | NA | NA | + | NA | Gamma  (log) | 11 | -23.5 | 23.68 | 0 |
| SC16 | + | + | + | + | NA | Gamma  (log) | 11 | -23.5 | 23.68 | 0 |
| SC17 | NA | NA | NA | + | NA | Gamma  (log) | 11 | -23.8 | 23.39 | 0 |
| SC18 | + | + | + | + | NA | Gamma  (log) | 11 | -23.6 | 23.62 | 0 |
| SC19 | NA | NA | NA | + | NA | Gamma  (log) | 11 | -23.6 | 23.62 | 0 |

***4. Color learning assay:*** To determine whether there was a difference between the color learning response (i.e., positive or negative response) as a function rearing groups (QR and WR), we carried out binomial GLMs to determine whether response during training procedure (i.e., "color training") and response after bees were trained (i.e., "color learning") were affected by predictor variables. We performed Binomial GLMMs with a binomial response ("positive" (1) or "negative" (0)) for each situation (training and learning) and the predictor factors as rearing history, body size, and developmental duration. We initially ran a null model without these factors and other models including these factors and interaction terms. For all analyses, we included colony source for queens and individual nests as random factors. All statistics for the model selection are summarized in Table S3.

**Table S3.** Selection of the best-fitting model explaining the learning assays. The models included rearing history (R; *i.e.,* rearing by queen or workers), body size (B), developmental duration (D) as the fixed factors, and source colony for queens and individual nest as the random factors. The best model fitting our data was selected based on the Akaike’s Information Criterion (AIC). (+) parameters included in the model, and NA, not included parameters. The selected best model is given in bold.

| **Color Training** | | | | | | | | | | |
| --- | --- | --- | --- | --- | --- | --- | --- | --- | --- | --- |
| **Model** | **R** | **B** | **D** | **S:D** | **T:S:D** | **family** | **df** | **AICc** | **delta** | **weight** |
| Null | NA | NA | NA | NA | NA | Binomial  (logit) | 3 | 57.3 | 0.92 | 0.195 |
| L1 | + | NA | NA | NA | NA | Binomial  (logit) | 4 | 59.3 | 2.96 | 0.07 |
| **L2** | **NA** | **+** | **NA** | **NA** | **NA** | **Binomial**  **(logit)** | **4** | **56.4** | **0** | **0.308** |
| L3 | NA | NA | + | NA | NA | Binomial  (logit) | 4 | 59.6 | 3.26 | 0.06 |
| L4 | + | + | + | NA | NA | Binomial  (logit) | 6 | 60.5 | 4.11 | 0.04 |
| L5 | + | NA | NA | + | NA | Binomial  (logit) | 7 | 63 | 6.58 | 0.011 |
| L6 | + | + | NA | NA | NA | Binomial  (logit) | 5 | 58.7 | 2.29 | 0.098 |
| L7 | + | NA | + | NA | NA | Binomial  (logit) | 5 | 61.7 | 5.35 | 0.021 |
| L8 | NA | + | + | NA | NA | Binomial  (logit) | 5 | 58 | 1.58 | 0.14 |
| L9 | NA | NA | NA | + | NA | Binomial  (logit) | 6 | 60.4 | 3.97 | 0.042 |
| L10 | + | + | + | + | NA | Binomial  (logit) | 7 | 63 | 6.58 | 0.011 |
| L11 | NA | NA | NA | + | NA | Binomial  (logit) | 10 | 69.6 | 13.18 | 0 |
| L12 | + | + | + | + | NA | Binomial  (logit) | 10 | 69.6 | 13.18 | 0 |
| L13 | NA | NA | NA | + | NA | Binomial  (logit) | 10 | 69.6 | 13.18 | 0 |
| L14 | + | + | + | + | NA | Binomial  (logit) | 10 | 69.6 | 13.18 | 0 |
| L15 | NA | NA | NA | + | NA | Binomial  (logit) | 10 | 69.6 | 13.18 | 0 |
| L16 | + | + | + | + | NA | Binomial  (logit) | 10 | 69.6 | 13.18 | 0 |
| L17 | NA | NA | NA | + | NA | Binomial  (logit) | 10 | 69.6 | 13.18 | 0 |
| L18 | + | + | + | + | NA | Binomial  (logit) | 10 | 69.6 | 13.18 | 0 |
| L19 | NA | NA | NA | + | NA | Binomial  (logit) | 10 | 69.6 | 13.18 | 0 |
| **Color Learning** | | | | | | | | | | |
| **Model** | **R** | **B** | **D** | **S:D** | **T:S:D** | **family** | **df** | **AICc** | **delta** | **weight** |
| **Null** | **NA** | **NA** | **NA** | **NA** | **NA** | **Binomial**  **(logit)** | **3** | **56.9** | **0** | **0.389** |
| Lt1 | + | NA | NA | NA | NA | Binomial  (logit) | 4 | 58.8 | 1.87 | 0.153 |
| Lt2 | NA | + | NA | NA | NA | Binomial  (logit) | 4 | 59.3 | 2.39 | 0.118 |
| Lt3 | NA | NA | + | NA | NA | Binomial  (logit) | 4 | 59 | 2.07 | 0.138 |
| Lt4 | + | + | + | NA | NA | Binomial  (logit) | 6 | 62.9 | 6.02 | 0.019 |
| Lt5 | + | NA | NA | + | NA | Binomial  (logit) | 7 | 65.5 | 8.56 | 0.005 |
| Lt6 | + | + | NA | NA | NA | Binomial  (logit) | 5 | 61 | 4.07 | 0.051 |
| Lt7 | + | NA | + | NA | NA | Binomial  (logit) | 5 | 60.4 | 3.46 | 0.069 |
| Lt8 | NA | + | + | NA | NA | Binomial  (logit) | 5 | 61.5 | 4.64 | 0.038 |
| Lt9 | NA | NA | NA | + | NA | Binomial  (logit) | 6 | 63.8 | 6.88 | 0.012 |
| Lt10 | + | + | + | + | NA | Binomial  (logit) | 7 | 65.5 | 8.56 | 0.005 |
| Lt11 | NA | NA | NA | + | NA | Binomial  (logit) | 10 | 72.6 | 15.66 | 0 |
| Lt12 | + | + | + | + | NA | Binomial  (logit) | 10 | 72.6 | 15.66 | 0 |
| Lt13 | NA | NA | NA | + | NA | Binomial  (logit) | 10 | 72.6 | 15.66 | 0 |
| Lt14 | + | + | + | + | NA | Binomial  (logit) | 10 | 72.6 | 15.66 | 0 |
| Lt15 | NA | NA | NA | + | NA | Binomial  (logit) | 10 | 72.6 | 15.66 | 0 |
| Lt16 | + | + | + | + | NA | Binomial  (logit) | 10 | 72.6 | 15.66 | 0 |
| Lt17 | NA | NA | NA | + | NA | Binomial  (logit) | 10 | 72.6 | 15.66 | 0 |
| Lt18 | + | + | + | + | NA | Binomial  (logit) | 10 | 72.6 | 15.66 | 0 |
| Lt19 | NA | NA | NA | + | NA | Binomial  (logit) | 10 | 72.6 | 15.66 | 0 |

***5. Starvation resistance*:** To determine whether there was a difference between the starvation resistance as a function to rearing groups (QR and WR), we performed the Mann-Whitney U-test. We then carried out Poison GLMMs to determine whether starvation resistance was determined by one of our predictor variables (rearing history, developmental duration and/or body size). We initially ran a null model without these factors and other models including these factors and interaction terms. We initially ran a null model without these factors and other models including these factors and interaction terms. For all analyses, we included colony source for queens, individual nests and behavioral assays (sucrose and learning assays) as random factors. All statistics for the model selection are summarized in Table S4.

**Table S4.** Selection of the best-fitting model explaining the starvation resistance assays. The models included rearing history (R; *i.e.,* rearing by queen or workers), body size (B), developmental duration (D) as the fixed factors, and source colony for queens, individual nest and behavioral assays as the random factors. The best model fitting our data was selected based on the Akaike’s Information Criterion (AIC). (+) parameters included in the model, and NA, not included parameters. The selected best model is given in bold.

| **Starvation resistance** | | | | | | | | | | |
| --- | --- | --- | --- | --- | --- | --- | --- | --- | --- | --- |
| **Model** | **R** | **B** | **D** | **S:D** | **T:S:D** | **family** | **df** | **AICc** | **delta** | **weight** |
| Null | NA | NA | NA | NA | NA | Gamma  (log) | 5 | 644.2 | 1.61 | 0.134 |
| S1 | + | NA | NA | NA | NA | Gamma  (log) | 6 | 643.5 | 0.89 | 0.191 |
| S2 | NA | + | NA | NA | NA | Gamma  (log) | 6 | 645.4 | 2.79 | 0.074 |
| S3 | NA | NA | + | NA | NA | Gamma  (log) | 6 | 646.4 | 3.86 | 0.043 |
| S4 | + | + | + | NA | NA | Gamma  (log) | 8 | 644.9 | 2.3 | 0.094 |
| S5 | + | NA | NA | + | NA | Gamma  (log) | 9 | 647.1 | 4.51 | 0.031 |
| **S6** | **+** | **+** | **NA** | **NA** | **NA** | **Gamma**  **(log)** | **7** | **642.6** | **0** | **0.299** |
| S7 | + | NA | + | NA | NA | Gamma  (log) | 7 | 645.6 | 2.98 | 0.067 |
| S8 | NA | + | + | NA | NA | Gamma  (log) | 7 | 648 | 5.39 | 0.02 |
| S9 | NA | NA | NA | + | NA | Gamma  (log) | 8 | 649.8 | 7.21 | 0.008 |
| S10 | + | + | + | + | NA | Gamma  (log) | 9 | 647.1 | 4.51 | 0.031 |
| S11 | NA | NA | NA | + | NA | Gamma  (log) | 12 | 654.6 | 12.01 | 0.001 |
| S12 | + | + | + | + | NA | Gamma  (log) | 12 | 654.6 | 12.02 | 0.001 |
| S13 | NA | NA | NA | + | NA | Gamma  (log) | 12 | 654.6 | 12.01 | 0.001 |
| S14 | + | + | + | + | NA | Gamma  (log) | 12 | 654.6 | 12.01 | 0.001 |
| S15 | NA | NA | NA | + | NA | Gamma  (log) | 12 | 654.6 | 12.01 | 0.001 |
| S16 | + | + | + | + | NA | Gamma  (log) | 12 | 654.6 | 12.01 | 0.001 |
| S17 | NA | NA | NA | + | NA | Gamma  (log) | 12 | 654.6 | 12.02 | 0.001 |
| S18 | + | + | + | + | NA | Gamma  (log) | 12 | 654.6 | 12.02 | 0.001 |
| S19 | NA | NA | NA | + | NA | Gamma  (log) | 12 | 654.6 | 12.02 | 0.001 |

**RESULTS**

**(a) Among brood cohorts and care-giver identity variation in developmental duration, body size, starvation resistance.**

We also explored variability in developmental durations, body size, and starvation resistance responses within brood cohorts and between rearing history groups using Levene's test (for non-parametric data), where the null hypothesis is that variance is equal.

With respect to body size, there was no difference detected in the amount of variation observed in the QR versus WR nests (Levene's test: F= 0.185, *p* = 0.668) or between brood from different nests (Levene's test: F= 1.305, *p* = 0.207). There was a significant difference in the amount of variation observed in developmental durations of bees from QR versus WR nests (Levene's test: F= 11.34, *p* = 0.001), with more variation in the QR nests (variance for QR =  21.446, WR =  5.793). Significant differences in variation were also detected between nests (Levene's test: F= 1.976, *p* = 0.020).

There was significant difference in variation in starvation resistance between offspring from QR and WR nests (Levene's test: F= 3.997, *p* = 0.048) or no significance between brood from different nests (Levene's test: F= 1.376, *p* = 0.187).

**(b) Sucrose responsiveness assay**


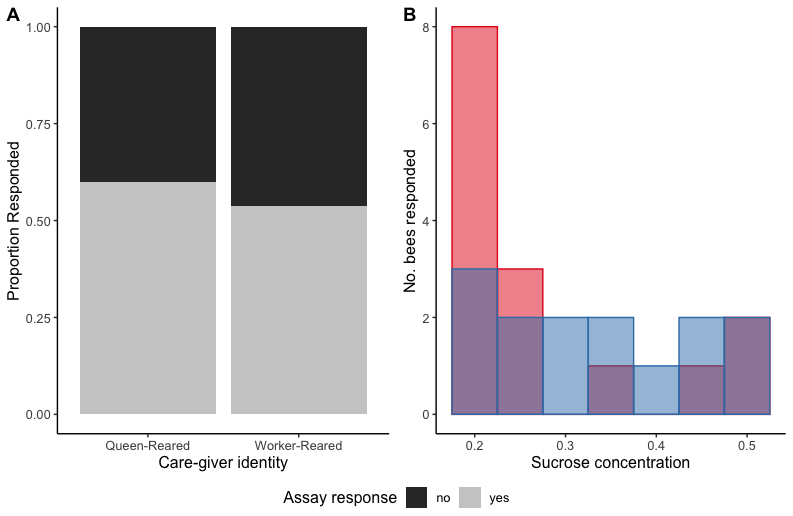


**Figure S3 | Effects of care-giver identity on sucrose responsiveness.** Young nests were manipulated such that the first cohort of female brood was either reared solely by a queen (hereafter, Queen-Reared or “QR” nests) or by a small cohort of workers (a set of five workers; Worker-Reared or “WR” nests). **(A)** The proportion of bees that responded in the sucrose assay (to any concentration) as a function of care-giver identity. **(B)** Sucrose responsiveness according to sucrose concentration, as a function of care-giver identity. Additional statistical information (including results of GLMMs) is provided in Table 2 and the electronic supplementary material.

**(c) Color learning assay**

***
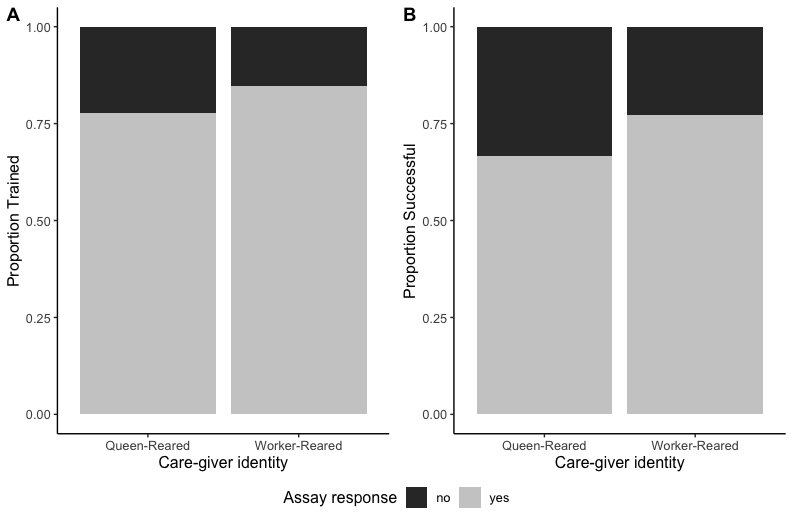
***

**Figure S4 | Effects of care-giver identity on color learning. (A)** The proportion of bees that were trained in the color learning assay as a function of rearing history. Young nests were manipulated such that the first cohort of female brood was either reared solely by a queen (hereafter, Queen-Reared or “QR” nests) or by a small cohort of workers (a set of five workers; Worker-Reared or “WR” nests). **(B)** The proportion of bees showing color learning as a function of care-giver identity. Additional statistical information (including results of GLMMs) is provided in Table 2 and the electronic supplementary material.

**REFERENCE**

1. Muth F, Cooper TR, Bonilla RF, Leonard AS. A novel protocol for studying bee cognition in the wild. Carvalheiro L, editor. Methods Ecol Evol. 2018;9(1):78–87. DOI: 10.1111/2041-210X.12852

2. Bates D, Mächler M, Bolker B, Walker S. Fitting Linear Mixed-Effects Models using lme4. Journal of Statistical Software. 2014:67, 1–48. Available from: http://arxiv.org/abs/1406.5823
